# Staples Won the Bet

**Published:** 1889-10

**Authors:** 


### STAPLES WON THE BET.

"THE RULING PASSION STRONG IN DEATH."

Some months ago two consumptives in the last stages of the disease lay dying on cots in close proximity to each other in the city hospital. Both victims were sports, who by dissipation had contracted phthisis in its most aggravated form. One was known as Bill Cunningham, a young gambler who had enjoyed the reputation among his class of being a reckless bettor on the turn of a card. The other was an English sailor named Staples, whose sole passion was to wager whatever he possessed in support of any opinion which he might express. As they lay on their cots, haggard and hollow-eyed and gasping for breath, they daily wasted the remnant of their vital forces in bantering one another about their appearance.

"I say, Staples," said Cunningham one morning in a voice scarcely above a hoarse whisper, "you're looking blue; better brace up, old man."

Staples, who really seemed to be a dead man as he lay almost breathless with his glazed eyes half open and mouth widely distended, pulled himself together with an indignant jerk and made an attempt to raise himself upon his arm.

"Billy," said he, "you're wrong. To prove it I'll bet you a dollar, the size of my pot, that I'll outlive ye."

"I'll see that bet," replied Billy.

An attendant was chosen as stakeholder and the money (all they possessed) was placed in his hands. Then began the struggle of these men to see who could retain the spark of life longest. At a distance of five feet the two dying men glanced at one another, each eager to show the other that his stock of vitality was the greater. Cunningham battled bravely, but he was the first to show signs of weakening. He finally resumed his old position, but it could be seen that his respiratory action was failing. Suddenly he gave one great gasp, and with that sigh the spark of his life, prematurely cut off, was extinguished.

"I've won the bet," said Staples, as he took the stake money with a gratified smile.

Cunningham's body was at once removed to the hospital morgue. The attendant had followed the cortege to the door and returned immediately to Staple's cot. Scarcely five minutes had possed since Cunningham had expired, but when the attendant glanced at Staples he saw that he, too, was dead. The last pot which he had raked in was clasped in his right hand. The grip was vise-like, and an instrument was employed to remove the silver from the stiffened unwilling fingers. —*San Francisco Chronicle.*

